# Multiyear Baleen Endocrine Profiles Suggest a Longer Estimated Gestation in Southern Right Whales (*Eubalaena australis*)

**DOI:** 10.1002/ece3.71528

**Published:** 2025-06-17

**Authors:** Loraine Shuttleworth, Andre Ganswindt, Kathleen E. Hunt, Alejandro Fernández Ajó, Estefan Pieterse, S. Mduduzi Seakamela, Chantel Schoeman, Els Vermeulen

**Affiliations:** ^1^ Faculty of Natural and Agricultural Sciences, Mammal Research Institute University of Pretoria Pretoria South Africa; ^2^ Smithsonian‐Mason School of Conservation Front Royal Virginia USA; ^3^ Geospatial Ecology of Marine Megafauna Lab, Department of Fisheries and Wildlife, Hatfield Marine Science Center, Marine Mammal Institute Oregon State University Newport Oregon USA; ^4^ Instituto de Conservación de Ballenas Ciudad Autonoma de Buenos Aires Argentina; ^5^ Department of Forestry, Fisheries and the Environment, Oceans and Coasts branch Cape Town South Africa

## Abstract

Gestation length is a key reproductive parameter influencing fecundity, population growth rates, and the recovery potential of baleen whales. However, direct knowledge of the gestation length in these large mammals remains limited, primarily inferred from whaling and observational data. Over the past decade, southern right whales have experienced a decline in reproductive success, likely linked to climate‐change‐induced shifts in foraging conditions. Understanding the population‐level consequences of these changes requires detailed longitudinal reproductive data. This study analyzes multiyear steroid hormone profiles in the baleen of adult female southern right whales stranded along the South African coast. Results show an extended hormonal pattern characterized by two peaks in progestogens between 20 and 25 months—suggesting putative pregnancies lasting substantially longer than previous estimates. Sharp estrogen peaks during periods of elevated progestogen phases may indicate hormonal regulation of myometrial contractions at birth. A positive correlation between progestogens and glucocorticoids suggests a role for glucocorticoids in pregnancy maintenance, while androgens provide limited insight into female reproduction in this species. These findings imply a longer‐than‐expected gestation period for southern right whales and potentially across the balaenid family. This has important implications for understanding the timing and location of conception, relevant for conservation management strategies. Multipopulation studies alongside individual sighting histories are recommended to refine our understanding of southern right whale reproduction further.

## Graphical abstract

Multiyear profiles of progesterone and estrogen in the baleen of adult female southern right whales indicate progesterone elevations lasting on average between 20 and 25 months, indicative of pregnancies longer than previously estimated. These data are in line with endocrine profiles in baleen of other Balaenid whales incl. North Atlantic right whale (NARW) and Bowhead whale. Data have implications on the timing and location of conception relevant for conservation management strategies.

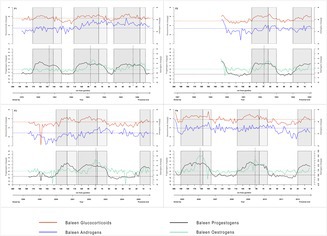

## 1 Introduction

The study of reproductive biology is essential for understanding species' reproductive strategies, managing wildlife populations, improving conservation efforts, and assessing the impacts of environmental changes on reproductive success and population sustainability. As a critical aspect of reproduction, gestation length is one of the key parameters influencing fecundity, population growth rates, and recovery potential subsequent to population declines. In general, the length of gestation in mammals is influenced by the degree of precociality (Sacher and Staffeldt 2018), body size (Martin and MacLarnon 1985), and metabolic rate (Blueweiss et al. 1978). In K‐selected species like large mammals, comparatively long gestation periods that result in few young per birth event are more common and subsequently lead to low reproductive rates, making these species particularly vulnerable to population declines and slow recoveries (Hayssen 1993). This is evident in baleen whales, among the largest animals to have ever lived and a well‐known example of K‐selected species (Estes 1979). Many baleen whale populations were hunted to near extinction in the 19th and 20th centuries and are still recovering (e.g., Bamford et al. 2023; Brandão et al. 2023; Gibbs et al. 2018; Jackson et al. 2020; Zerbini et al. 2019).

Knowledge of gestation length of baleen whales, especially Balaenids, is limited. Nonetheless, accurate knowledge of this parameter is essential for a better understanding of reproductive cycles and developing realistic population models to forecast population trends, which in turn guide conservation strategies. Current knowledge of gestation length of Balaenid whales is inferred from whaling data, estimated based on catch date, location, and fetal length (e.g., Best 1994; Chittleborough 1957; Lockyer 1984; Reese et al. 2001). Observational data on intercalving intervals have also been used to infer gestation length, which in many baleen whales is assumed to be tightly linked to the annual migrations between low‐latitude mating and calving grounds and high‐latitude feeding grounds (e.g., Best 1994; Lockyer 1984). This is because baleen whales must balance energy acquisition during seasonal prey availability with the need to give birth in a location suitable for calf survival (Eichenberger et al. 2023). As a result, migration timing is closely linked to reproductive cycles, ensuring conception and birth align with optimal environmental conditions for both maternal condition and calf rearing (Eichenberger et al. 2023). However, minimal data exist on the reproductive physiology of baleen whales, with only a handful of studies conducted so far (e.g., Dalle Luche et al. 2020; Hunt et al. 2019; Melica et al. 2021). These studies have been limited in their ability to provide information regarding important reproductive events, including ovulation, fertilization, or implantation, due to the inability to apply methods such as ultrasound, which is routinely used to study captive toothed whales (Brook 2001). Of these existing studies, very few provide longitudinal physiological information that can inform estimates of gestation length, for example, from the duration of elevated progestogens across time in the same individuals. More specifically, they have been limited to three species: the North Atlantic right whale (NARW; *Eubalaena glacialis* ; Hunt et al. 2016; Lysiak et al. 2018), the bowhead whale ( *Balaena mysticetus* ; Lysiak et al. 2023) and the humpback whale (*Megaptera novaeangliae*; Lowe et al. 2022). These studies indicate that endocrine research, especially longitudinal research, may prove informative in other species as well.

Southern right whales ( *Eubalaena australis* ; SRWs) were severely hunted to near extinction, with only a few hundred individuals remaining globally by the 1920s (Jackson et al. 2008). Their population was estimated at ~13,600 individuals in 2009, with the species distributed over 12 winter calving grounds in the Southern Hemisphere (IWC 2013). Due to their site fidelity to coastal calving grounds, the species has been studied extensively for the past five decades in several regions, providing a wealth of data on population demographics post‐whaling (e.g., Bannister 2001; Best et al. 2001; Brandão et al. 2023; Burnell 2001; Carroll et al. 2011; Charlton et al. 2019; Payne 1986; Stamation et al. 2020; Watson et al. 2021). These data revealed that several SRW populations are recovering at an approximate rate of 7% per year, thought to be the maximum biologically possible for the species (e.g., Best et al. 2001; Cooke et al. 2001), although some populations remain critically endangered (Stamation et al. 2020; Vernazzani et al. 2014). Decades of observational data have clarified age at first parturition and indicated that the majority of healthy females follow a typical 3‐year calving cycle, which has been interpreted as consisting of 1 year of gestation, 1 year of lactation, and 1 year of “rest” (a nonpregnant and nonlactating phase, during which the female whale recovers body condition) (Best 1994; Best et al. 2001; Cooke et al. 2001). Current population models are based on these reproductive parameters (Brandão et al. 2023; Cooke et al. 2001). Despite this steady recovery, the South African population has undergone demographic changes over the past decade, including an increasing number of females calving at 4‐ and 5‐year intervals (Brandão et al. 2023). These changes have been linked to decreased maternal body condition (Vermeulen, Thavar, et al. 2023) as well as a change in foraging strategy (van den Berg et al. 2021), both likely a result of decreased food availability in Southern Ocean foraging grounds (Germishuizen et al. 2024). Similarly, the SRW population in the Southwest Atlantic has also been shown to be affected by climate change in its reproductive parameters and survival (Agrelo et al. 2021; Leaper et al. 2006; Seyboth et al. 2016). These population‐level changes underscore the need for a deeper assessment of reproductive biology to better understand the physiological responses and trade‐offs associated with climate‐driven shifts in foraging conditions. As the quantification of steroid hormones has become a more widely utilized tool for investigating the reproductive biology of various species (Ganswindt et al. 2012; Hunt et al. 2013), this study investigates multiyear longitudinal hormone profiles derived from the baleen plates of stranded adult female SRWs to obtain detailed information on the endocrine correlates of SRW reproduction. Elevated progestogens are indicative of pregnancy in all Balaenidae yet examined (Hunt et al. 2016; Inoue et al. 2019; Kellar et al. 2013), as in most mammals. Thus, progestogen profiles assessed here in detail were used as a method to investigate the gestation period of SRWs. Baleen was used as a sample matrix, as it differs from other matrices in that it grows continuously, simultaneously incorporating circulating hormones and, in this way, preserves an unbroken record of endocrine data spanning multiple years (Hunt et al. 2016). Baleen from SRWs is particularly suitable for longitudinal studies of life history events due to its exceptional length and established growth rate in adults (Best and Schell 1996). Specifically, in adult female SRWs, a single baleen specimen represents a continuous endocrine record of approximately the last 7 years of the whale's life. To our knowledge, this is the first study to assess endocrine patterns in SRW baleen.

Our sample size is limited to four individuals, and thus the generalizability of our results may be limited. This low *n* of individuals is typical of baleen‐hormone research, partly due to logistical constraints imposed by the high number of consecutive sub‐samples for each individual, but also due to the low likelihood of finding an adult whale that has stranded in a location and position from which the baleen can be excised. Nonetheless, such small‐*n* studies can provide informative data, particularly in cases in which basic biological information is lacking on the research question—such as is the case for Balaenidae gestation length and mysticete biology generally—and in cases in which the individual datasets provide repeated sampling of the same individuals over long periods of time (Clutton‐Brock and Sheldon 2010; Schork et al. 2023), as is the case here. Our primary study objectives include: (1) determining whether steroid hormones are detectable and quantifiable along the full length of adult female SRW baleen, (2) constructing time‐series profiles of steroid hormones, (3) evaluating the duration of progestogen elevations to estimate gestation length, acknowledging the limitations of using endocrine data without direct evidence of the exact timing of conception and parturition, and (4) evaluating intercalving interval from progestogen profiles as opposed to calf sightings. We predict that baleen from adult female SRWs will reveal periodic elevations in progestogens that last slightly longer than the current accepted SRW gestation length of 12–13 months (Best 1994), but that a 3‐year intercalving interval will still be observed.

## 2 Methods

### 2.1 Study Animals

This study used baleen plates from four adult female SRWs that stranded along the coastline of South Africa between 1987 and 2013 (Table 1) under South African government permit RES2021‐18 and University of Pretoria Ethics Permit number NAS224/2021.

**TABLE 1:** Details regarding the animals and their respective baleen plates used as samples for this study.

Animal ID	Animal length (m)	Year stranded	Total baleen length (m)	Baleen length from gumline (m)	Number of samples	Approximate time frame represented
F1	13.73	1987	2.29	2.08	187	~8 years
F2	15.5	1995	1.50	1.36	132	~6 years
F3	14.65	2006	2.10	1.90	178	~7 years
F4	14.3	2013	2.14	1.98	198	~8 years

These four females were chosen for the study as they were the only specimens available from females ≥ 12.4 m in total body length (confirmed at stranding), a widely accepted size threshold for adult (and thus reproductively mature) females (Best and Rüther 1992). Due to the lack of associated photo‐identification data, none of these four individuals have a known sighting history, and the reproductive history of all four individuals was unknown. Classic biological validations are, therefore, not yet possible for this population (i.e., confirmation that endocrine data agree with independent observational confirmation of physiological status of the whale). However, based on consistent data from other balaenid species demonstrating close matches of baleen progestogen patterns to pregnancy history (see Hunt et al. 2014, 2016; Lysiak et al. 2018), we regard it as a reasonable assumption that any region with sustained high progestogen in SRW baleen would be indicative of a pregnancy. The baleen plates were airdried and stored in the archives of the Iziko South African Museum and the Department of Forestry, Fisheries and the Environment, South Africa, where they were sampled.

### 2.2 Sample Collection

**FIGURE 1:**
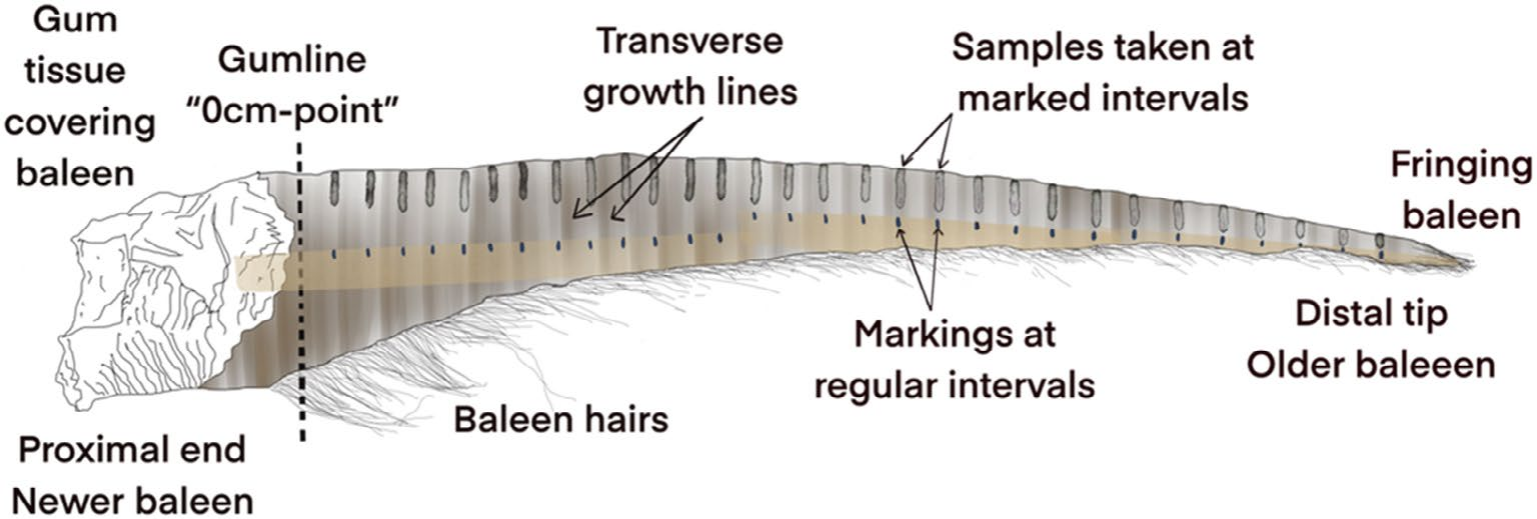
A schematic diagram illustrating the methodology used to sample each baleen plate.

The longest baleen plate available per individual was chosen for sampling, following the sampling protocol of Hunt et al. (2018). In brief, each baleen plate was first cleaned with 70% ethanol and, if necessary, brushed to remove any sand or debris. Gloves were used to avoid possible contamination from hormones in human skin oils. Masking tape was then fixed to each baleen plate parallel to the labial edge and, using a flexible tape measure as a guide, markings were made on the masking tape at 2 cm intervals to indicate where samples would be taken (Figure 1). This interval was chosen based on the assumption that it would produce a temporal resolution of an estimated 27–34 days due to the adult SRW baleen growth rate (Best and Schell 1996). Markings began at the “0 cm‐point” which was allocated to the gumline (newer baleen) and continued until the distal tip (older baleen). Note that the baleen embedded within the gum, which would reflect approximately the last year of the whale's life, was not sampled.

A hand‐held flexible shaft engraving tool (Dremel 3000 with High Speed Cutter 191 attachment) was used to pulverize samples of baleen by grinding away at the plate at each marked interval. Care was taken to follow and never cross the transverse lines visible on the baleen, which are assumed to be baleen growth lines. Samples were only taken from solid baleen and not from the “hairs” that fringe the lingual border of the solid baleen plate, to account for the possibility that hormone extraction efficiency may differ markedly for hairs vs. solid baleen. Samples were also taken specifically from the outer layer (cortex) and not from the inner layer (medulla), as the adjoining cortex and medulla are now known to represent different dates of growth, with the cortex laid down before the medulla (Rita et al. 2019). Each sample was collected directly after pulverization in aluminum foil, weighed to ensure 0.20–0.30 g of baleen powder was collected, and then stored in a labeled paper envelope. The work surface was then blown with a hairdryer to remove excess baleen powder and avoid cross‐contamination. The baleen, work surface, equipment, and gloved hands were then cleaned with 70% ethanol before beginning pulverization of the next sample.

### 2.3 Steroid Extraction

Following Hunt et al. (2018), between 0.020 and 0.050 g of baleen powder from each sample was used for steroid extraction by adding 100% methanol. The amount of methanol added was adjusted according to the sample weight using 1 mL for samples < 0.032 g, 1.5 mL for samples between 0.032 and 0.042 g, and 2 mL for samples > 0.042 g. This process was designed to keep the solvent: sample ratio (mL methanol to g of baleen powder) within a relatively narrow range, as large deviations in the solvent: sample ratio can affect hormone extraction efficiency (Fernandez Ajo et al. 2022). Suspensions were vortexed for 2 h and then centrifuged for 15 min at 4000 *g*. Resulting supernatants were transferred into Eppendorf microcentrifuge tubes, sealed with parafilm, and stored at −20°C until analysis at the Endocrine Research Laboratory, University of Pretoria, South Africa.

### 2.4 Steroid Quantification

Steroid extracts were analyzed for baleen progestogen, estrogen, and androgen concentrations using in‐house enzyme immunoassays (EIAs) and detailed assay characteristics, including cross‐reactivities, can be found in Schwarzenberger et al. (1996) for the progestogen EIA, Palme and Möstl (1993) for the estrogen and androgen EIA used (Table 2). In brief, 50 mL aliquots of the respective standard dilution, quality controls, and diluted baleen extracts were pipetted in duplicate into 96‐well microtiter plate wells. Then, 50 mL of biotinylated steroid label and antiserum were added, and the plates were incubated overnight at 4°C. Following incubation, the plates were washed four times, and 150 mL (20 ng) of streptavidin‐peroxidase was added to each well. Following incubation in the dark for 30 min, plates were washed again before 150 mL peroxidase substrate solution was added, and plates were further incubated for 30–60 min. The reaction was terminated by adding 50 mL of 4 N H_2_SO_4_, and the absorbance was measured at 450 nm. Samples were re‐analyzed if the optical density of the duplicates differed by more than 10%. Classic biological validations were not possible for the reproductive hormone assays; however, it is established in previous literature that progesterone, testosterone, and estradiol assays do produce data that agree with the known pregnancy history of females in closely related species (Hunt et al. 2014). Furthermore, preliminary mass spectrometry analysis indicates that these reproductive hormones are indeed present in baleen powder of multiple mysticete species (Hunt, unpub. data). The sensitivities of the assays are listed in Table 2. Intra‐assay and interassay coefficients of variation (CV) have been determined by repeated measurements of high (*n* = 18 [Intra] 57–60 [Inter] per assay) and low (*n* = 17 [Intra] 57–60 [Inter] per assay) quality controls generated from the respective standard stock solutions. Assay parallelism for the progestogen, estrogen, and androgen assays was assessed using pooled samples (1–5 samples per pool), and resulted in displacement curves that were parallel to the respective standard curves and had a relative variation in the slope of the trendlines of < 3%, < 5%, and < 2% for the EIAs, respectively (Figure S1, Figures S2 and S3). Additional validations were performed for the glucocorticoid assay because the primary glucocorticoid has not yet been determined for mysticetes. Cortisol and corticosterone assays, which have previously been validated for SRWs (Fernández Ajó et al. 2018), both pass parallelism validations but produce different biologically plausible patterns across time, with species‐specific variation observed in hormone abundance (Hunt et al. 2018). To identify the most suitable EIA for assessing baleen glucocorticoids, the following five different EIAs were used to compare samples from females presumed to be pregnant with samples from presumably nonpregnant females and with samples from males, as pregnancy was considered a stressor which could be used to biologically validate the suitability of a glucocorticoid assay: (1) a 11‐Oxoaetiocholanolone I EIA (detecting 11,17 dioxoandrostanes), (2) a 11‐Oxoaetiocholanolone II EIA (detecting fGCMs with a 5β‐3α‐ol‐11‐one structure), (3) a 5α‐pregnane‐3β,11β,21‐triol‐20‐one EIA (detecting fGCMs with a 5α‐3β,11β‐diol structure), (4) a Cortisol EIA, and (5) a corticosterone EIA. Detailed assay characteristics, including cross‐reactivities, can be found in Palme and Mostl (1997) for the 11‐oxoetiocholanolone I, cortisol, and corticosterone EIAs, Möstl et al. (2002) for the 11‐oxoetiocholanolone II EIA, and Touma et al. (2003) for the 5α‐pregnane‐3β,11β,21‐triol‐20‐one EIA (Table S1).

**TABLE 2:** Specifications of the EIAs used to quantify steroid hormones in baleen powder.

EIA name	Label	Antibody raised against	Standard	Sensitivity (ng/g dry weight)	Intra‐assay coefficient of variation (%)		Interassay coefficient of variation (%)	
					[High] control	[Low] control	[High] control	[Low] control
Progesterone (Schwarzenberger et al. 1996)	5α‐pregnane‐3β‐ol‐20‐one‐3HS:DADOO‐biotin	5β‐pregnane‐3α‐ol‐20‐one‐3HS:BSA	Progesterone (5α‐pregnan‐3β‐ol‐20‐one)	12.8	5.64	6.12	13.02	13.15
Oestrone (Palme and Möstl 1993)	17β‐oestradiol‐17‐glucosiduronate: DADOO‐biotin	17β‐oestradiol‐17‐HS:BSA	Oestrone	0.32	5.85	6.27	8.76	9.05
Testosterone (Palme and Möstl 1993)	5α‐androstane‐3β,17β‐diol‐3‐HS:DADOO‐biotin	Testosterone‐3‐CMO:BSA	Testosterone (17β‐Hydroxy‐3‐oxo‐4‐androstene)	1.60	6.58	7.597	7.77	11.45
Corticosterone (Palme and Mostl 1997)	Cortisol‐3‐CMO‐DADOO‐biotin	Corticosterone‐3‐CMO‐BSA	Corticosterone (4‐pregnene‐11β,21‐diol‐3,20‐dione)	3.2	2.81	6.31	7.19	9.58

Abbreviations: BSA, bovine serum albumin; CMO, carboxymethyloxime; DADOO‐biotin, *N*‐biotinyl‐1,8‐diamino‐3,6‐dioxaoctane; HRP, horseradish peroxidase; HS, hemisuccinate.

The sensitivities of the assays are listed in Table S1. For determining the most suitable assay, intra‐assay and interassay CV has been determined by repeated measurements of high (*n* = 18 [Intra] 3–15 [Inter] per assay) and low (*n* = 17 [intra] 3–15 [inter] per assay) quality controls generated from the respective standard stock solutions, with concentrations located at around 20% (high) and 80% (low) of the linear range of the respective binominal standard curve (Table S1). The corticosterone assay appeared to be the most reliable of the tested EIAs for measuring glucocorticoid concentrations in SRW baleen, and respective interassay CVs were determined by using high‐ and low‐quality controls (*n* = 60 in both cases) (Table S1). A parallelism analysis was not required, as all samples have been measured in a 1/5 dilution. All assays were performed on microtiter plates following established protocols (Ganswindt et al. 2012) at the Endocrine Research Laboratory at the Faculty of Natural and Agricultural Sciences, University of Pretoria. As is common for nonliquid sample types (e.g., fur, feather, baleen), recovery tests (i.e., to measure extraction efficiency) were not performed due to the fact that added hormone cannot be bound into the particles of solid sample types in the same way as native hormone (i.e., extraction efficiency of added hormone is not informative about extraction efficiency of native hormone). Hence, extraction efficiency is unknown but is assumed to be similar across all samples. Our data analysis therefore focuses on relative patterns rather than absolute concentrations.

### 2.5 Data Analysis

An individual baseline concentration for each baleen plate and steroid class was calculated using an iterative approach where samples were arranged in descending order, and any samples greater than or equal to the mean +1.5 SD were excluded. This process was repeated until all remaining samples were below this value (Brown et al. 1999). Longitudinal baleen hormone concentrations and their respective baselines (see Table 3) were subsequently plotted for each individual and visually inspected for trends of elevated periods for each steroid class. Note that the calculated baseline values served as a general indicator to differentiate elevated from nonelevated periods; however, they should be considered an estimate, as smaller fluctuations may still hold biological relevance.

**TABLE 3:** Baseline hormone metabolite values calculated using an iterative approach, excluding values ≥ mean +1.5 SD (Brown et al. 1999).

	Baleen progestogens (μg/g)	Baleen estrogens (ng/g)	Baleen androgens (ng/g)	Baleen glucocorticoids (ng/g)
F1	0.2553	2.0160	26.8762	45.4774
F2	0.2161	2.1782	26.1093	33.2362
F3	0.2184	2.3037	21.5372	38.1532
F4	0.3218	2.2524	31.888	50.0961

Periods of elevated progestogen concentrations, defined as levels of at least 1.5 times the baseline value and sustained across at least three consecutive samples, were used to determine gestation length in SRWs. Elevated periods that included both the beginning and end of the high progestogen period were classed as “complete.” “Incomplete” progestogen elevations were those that were located at the proximal or distal ends of the baleen plate such that the beginning or end of the elevation was not recorded in the baleen, that is, duration could not be estimated. Complete elevations were further divided into different segments based on visible peaks. The first peak refers to the segment from the initial rise in progestogen concentration to its first maxima and subsequent decline, while the second peak begins when progestogen concentrations begin to rise again, reaching the final maxima and ending when values return to baseline. The duration of the segments was estimated by counting the number of samples within the segment and applying the estimated baleen growth rate in adult SRWs (27–34 days per sample) as established by Best and Schell (1996). Similarly, the duration of time spent at baseline was also calculated. Our method rests on assumptions of a constant rate of baleen growth and hormone incorporation into the baleen. To assess correlation between different hormones within and between individuals, a Spearman's correlation test was performed. All statistical analyses were performed in R using base R functions and the “ggcorrplot” package for visualization of correlation (R Core Team 2022).

## 3 Results

### 3.1 Baleen Hormone Concentrations

**FIGURE 2:**
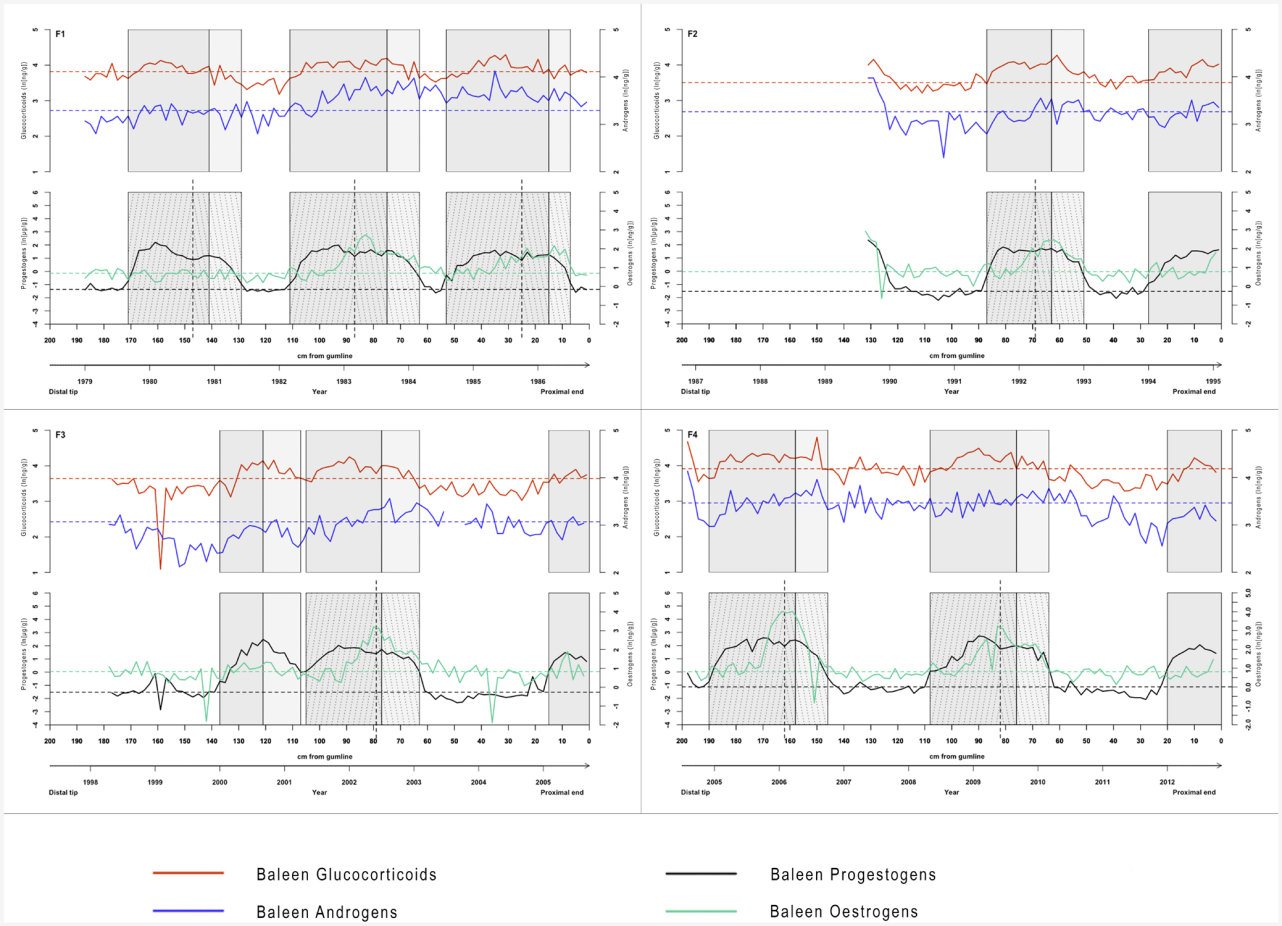
Longitudinal glucocorticoid (red), androgen (blue), progestogen (black) and, estrogen (green) profiles in baleen plates of individual adult female southern right whales. Note the logarithmic scale. The upper *x*‐axis depicts samples in cm from the gumline, while the lower *x*‐axis depicts an estimation of time in years (1 sample = 1 month). Horizontal dotted lines parallel to the *x*‐axis represent the baseline values of each hormone unique to each individual. Shaded areas highlight regions with elevated progestogen concentrations: Darker gray indicates the rise to the final maxima, and lighter gray shows the decline to baseline. Vertical dashed lines divide the elevation into two peaks, with forward‐slanted dashed lines marking Peak 1 and backward‐slanted dashed lines marking Peak 2. F1: *N* = 187 samples, F2: *N* = 66 samples, F3: *N* = 89 samples and F4: *N* = 99 samples.

Each baleen plate showed multiple periods of prolonged elevations in baleen progestogen concentrations (i.e., putative pregnancies) interspaced with periods of lower progestogen concentrations (putative nonpregnant periods; Figure 2). Across the four baleen plates, 11 prolonged elevations in progestogen concentration were observed in total. Three elevations per baleen specimen were observed for three individuals, while the fourth individual (F2, upper right in Figure 2), which had the shortest baleen, had only two progestogen elevations. This latter individual (F2) also had a few elevated points at the distal tip of the plate that may represent the very end of a prior pregnancy.

Eight progestogen elevations were classed as “complete” such that duration could be estimated. Seven of these eight had a bimodal pattern across time, with the second peak of the bimodal elevation always being lower than the first. The prolonged elevations had an average length of 21.75 ± 2.72 SD sample points (range: 16–25 sample points). When considering the estimated baleen growth rate of adult SRWs of 27–34 days for every 2 cm (Best and Schell 1996), the elevations lasted on average between 587 days (approximately 20 months) and 740 days (approximately 25 months) (Table 4). Most periods of elevated progestogen concentrations were separated from each other by intervals of low‐progestogen concentrations that differed in duration but lasted on average for 14.5 ± 7.4 SD sample points (range: 4–25 sample points), or between 392 days (approximately 13 months) and 493 days (approximately 16 months). However, one female (F3; lower left in Figure 2) had two elevations in very close succession with no low‐progestogen period separating them. The inferred total reproductive intervals (duration between the start of two consecutive progestogen elevations) varied but lasted an average of 33.9 ± 10.8 SD sample points (range: 16–47 sample points) or between 914 days (approximately 30 months/2.5 years) and 1151 days (approximately 38 months/3 years) (Table 4).

**TABLE 4:** The length of segments of baleen progestogen profiles reported as the number of samples taken at 2 cm intervals and converted to the number of months^a^.

	Elevation	Peak 1		Peak 2		Initial rise to final maxima		Decline from final maxima to baseline		Total elevation	Subsequent baseline	
		Samples	Months	Samples	Months	Samples	Months	Samples	Months	Months	Samples	Months
F1	1	13	11.7–14.7	9	8.1–10.2	16	14.4–18.1	6	5.4–6.8	19.8–24.9	8	7.2–9.1
	2	13	11.7–14.7	12	10.8–13.6	19	17.1–21.5	6	5.4–6.8	22.5–28.3	4	3.6–4.5
	3	15	13.5–17	9	8.1–10.2	20	18–22.7	4	3.6–4.5	21.6–27.2	—	—
F2	1	10	9–11.3	9	8.1–10.2	13	11.7–14.7	6	5.4–6.8	17.1–21.5	11	9.9–12.5
F3	1	16	14.4–18.1	—	—	9	8.1–10.2	7	5.6–7.9	14.4–18.1	—	—
	2	14	12.6–15.9	8	7.2–9.1	15	13.5–17	7	5.6–7.9	19.8–24.9	25	22.4–28.3
F4	1	15	13.5–17	8	7.2–9.1	17	15.3–19.3	6	5.4–6.8	20.7–26.1	18	16.2–20.4
	2	14	12.6–15.9	9	8.1–10.2	17	15.3–19.3	6	5.4–6.8	20.7–26.1	21	18.9–23.8

^a^ Based on the estimation of baleen growth rate at 2 cm per 27–34 days in adult southern right whales (Best and Schell 1996) and assuming 1 month = 30 days. Each bimodal elevation in progestogen concentration is composed of two peaks. Peak 1 refers to the segment from the initial rise in progestogen concentration to its first maxima and subsequent decline, while Peak 2 begins with the following rise in progestogen concentration, reaching the final maxima and ending when values return to baseline.

Near the second crest of the bimodal progestogen elevations, baleen estrogen concentrations often increased sharply between 4‐ and 24‐fold above baseline (Figure 2). No increase in estrogen concentration was observed during the first progestogen elevation of F1 or the unimodal progestogen elevation of F3. Values of baleen androgen concentrations elevated sporadically above baseline for short periods at a time; however, no recognizable pattern was visible (Figure 2). Periods of baseline baleen glucocorticoid levels were interspaced by multiple longer elevations in glucocorticoid concentrations. Elevated glucocorticoid concentrations mostly coincided with elevations in progestogen concentrations (Figure 2).

### 3.2 Correlation Between Hormone Metabolites

**FIGURE 3:**
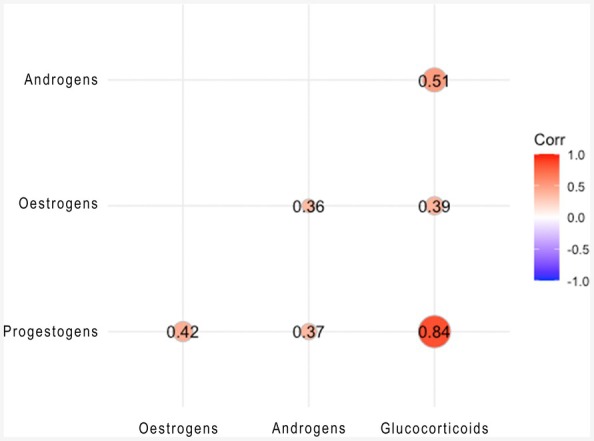
Plot depicting the correlation between baleen progestogen, estrogen, glucocorticoid, and androgen concentrations measured at 2 cm intervals in the baleen plates of four adult female southern right whales.

Positive correlations were observed between all hormones on both an individual level and across the combined sample set (Table 5; Figure 3). A strong positive correlation was observed between baleen progestogen and glucocorticoid concentrations, while correlations between other hormones ranged from weak to moderate. All correlations but one (F3, glucocorticoids vs. androgens) were statistically significant (*p* < 0.01).

**TABLE 5:** Results of Spearman's correlations of baleen progestogens, estrogens, glucocorticoids, and androgens, measured in the baleen plates of four adult female southern right whales combined, as well as in the individuals separately (F1–F4; statistical significance is indicated by bold).

Individual	Progestogens vs. estrogens	Progestogens vs. glucocorticoids	Progestogens vs. androgens	Estrogens vs. glucocorticoids	Estrogens vs. androgens	Glucocorticoids vs. androgens
Overall	*r* = 0.425	*r* = 0.836	*r* = 0.367	*r* = −0.389	*r* = 0.359	*r* = 0.512
	***p* < 0.01**	***p* < 0.01**	***p* < 0.01**	***p* < 0.01**	***p* < 0.01**	***p* < 0.01**
F1	*r* = 0.281	*r* = 0.768	*r* = 0.305	*r* = 0.363	*r* = 0.592	*r* = 0.376
	***p* < 0.01**	***p* < 0.01**	***p* < 0.01**	***p* < 0.01**	***p* < 0.01**	***p* < 0.01**
F2	*r* = 0.474	*r* = 0.903	*r* = 0.463	*r* = 0.440	*r* = 0.361	*r* = 0.448
	***p* < 0.01**	***p* < 0.01**	***p* < 0.01**	***p* < 0.01**	***p* < 0.01**	***p* < 0.01**
F3	*r* = 0.348	*r* = 0.855	*r* = 0.127	*r* = 0.347	*r* = −0.472	*r* = 0.234
	***p* < 0.01**	***p* < 0.01**	***p* < 0.01**	***p* < 0.01**	***p* < 0.01**	*p* = 0.029
F4	*r* = 0.564	*r* = 0.816	*r* = 0.383	*r* = 0.452	*r* = 0.299	*r* = 0.543
	***p* < 0.01**	***p* < 0.01**	***p* < 0.01**	***p* < 0.01**	***p* < 0.01**	***p* < 0.01**

## 4 Discussion

All four baleen plates used in this study revealed an uninterrupted longitudinal time series of the measured steroid hormones. Results indicate that elevations in progestogen concentrations lasted on average between 20 and 25 months. While this is considerably longer than the ~12–13 month gestation length that has long been assumed for Balaenidae (Best 1994; Nerini et al. 1984), it is consistent with recent observations of progestogen patterns in baleen of the other balaenid species such as NARWs (Hunt et al. 2016; Lysiak et al. 2018) and bowhead whales (Lysiak et al. 2023). Given that elevated progestogens are widely recognized as an indicator of gestation across mammals including cetaceans (Kellar et al. 2013; Hunt et al. 2014, 2016; Galligan et al. 2020; Lowe et al. 2022), these findings suggest a gestation period substantially longer than 1 year in duration, not only in SRWs but potentially across the balaenid family. They further support that patterns of reproductive steroids in keratin tissues can provide useful information on gestation length.

Previous estimates of SRW gestation length of approximately 12–13 months are based on fetal lengths derived from whaling data and an assumption of a continuous linear growth rate (Best 1994). That is, fetal lengths of mid‐gestation and late gestation of whales killed in different months of the year were compared to derive a fetal growth rate characteristic of late gestation, and this growth rate was then extrapolated backwards using linear regression analyses to estimate the date at which the zygote was formed, with the underlying assumption that fetal growth rate does not change during gestation (Best 1994). However, Best (1994) explicitly cautioned that basing estimates on a linear fetal growth rate could be inaccurate, and recommended further research into the issue. In fact, several authors have argued that fetal growth follows a curvilinear, rather than linear, pattern in baleen whales and that applying linear rates likely underestimates gestation length (e.g., Christiansen et al. 2014, 2022; Laws 1959; Reese et al. 2001; Rice and Wolman 1971). However, accurately determining gestation length in baleen whales remains challenging; while a newborn calf signals the end of gestation, identifying the start (conception) is difficult due to the inability to collect observational data. Classic methods of confirmation of fertilization and early gestation, such as uterine lavage of early embryos, ultrasound of early stages of pregnancy, or chorionic gonadotropin assays of blood samples, are not feasible in baleen whales. The incorporation of physiological data, particularly from baleen plates, which retain uninterrupted endocrine profiles over several years (Hunt et al. 2014), therefore offers valuable insight.

The progestogen profiles observed in this study showed predominantly a bimodal pattern, with elevated estrogen levels during the second peak, similar to findings in NARWs (Hunt et al. 2016; Lysiak et al. 2018) and bowhead whales (Hunt et al. 2014; Lysiak et al. 2023). In NARWs, Hunt et al. (2016) identified periods of elevated progestogens in baleen corresponding to an estimated gestation length of 15–18 months. For bowhead whales, Lysiak et al. (2023) suggested that, based on the absence of intermediate‐sized fetuses in the spring subsistence whaling data, the prolonged 23‐month period of progestogen elevation likely does not correspond to a 2‐year gestation. Instead, they proposed two hypotheses to explain the bimodal progestogen profile. First, they posited that the initial progestogen peak could be associated with delayed fertilization or implantation, polyestry, prolonged estrus, or a combination of these. True gestation would then only begin during the second peak, reported to last on average 13.6 months in bowhead whales (Lysiak et al. 2023). Under this interpretation, SRW gestation in the present study would last approximately 8.2–10.3 months, with progestogen levels remaining high for 12.3–15.5 months prior (average length of first progestogen peak). However, extended estrus or polyestry lasting several months seems unlikely for seasonal breeders like baleen whales, which must synchronize reproduction with migratory and feeding cycles (Corkeron and Connor 1999). In the case of delayed fertilization or embryonic diapause, which may provide ways of postcopulatory sexual selection in species that exhibit sperm competition like balaenids (Orr and Zuk 2014; Parker 1970), mating for conception would occur immediately postlactation, followed by a prolonged delay in fertilization or implantation. Although embryonic diapause is common in species that need to optimize the timing of birth and seasonal food availability (Renfree and Shaw 2000), there do not seem to be evolutionary or environmental pressures that would support the delay of fertilization and/or implantation in baleen whales with a full year.

Alternatively, Lysiak et al. (2023) suggested that gestation may commence at the point progestogens rise above baseline and end at the maxima of the second peak. In this case, the bimodal progestogen profile may reflect a luteal‐placental shift in progestogen production, common in many mammalian species (including marine mammals such as killer whales, *Orcinus orca* ; Duffield et al. 1995; Robeck et al. 2016), especially in species with gestations exceeding 151 days (Davies and Ryan 1972). This mechanism could explain the sharp decline in progestogens after parturition observed in a few NARWs (Hunt et al. 2016), although the hormonal half‐life in large whales would likely be extended (Lysiak et al. 2023). Additionally, the marked increase in estrogen during the second progestogen peak observed across all three balaenid species (NARWs: Lysiak et al. 2018; bowhead whales: Lysiak et al. 2023; SRWs: this study) could relate to myometrial contractions, as seen in killer whales (Robeck et al. 2016), humpback whales (Lowe et al. 2022), and artiodactyls (Challis 1971). Under this more likely scenario, gestation would last approximately 16 months for both bowhead whales (Lysiak et al. 2023) and SRWs (this study).

Gestation length in mammalian species generally correlates with body size (Martin and MacLarnon 1985) and metabolic rate (Blueweiss et al. 1978). However, baleen whales often deviate from predicted gestation lengths based solely on body mass, likely due to the constraints imposed by seasonal migrations (Lysiak et al. 2023). Within baleen whales, balaenids further differ significantly in reproductive strategies compared to rorquals (family Balaenopteridae), likely due to their lower metabolic rates. Balaenids' slow, energy‐efficient skim‐feeding behavior and thick blubber layer allow them to survive in nutrient‐scarce environments, resulting in lower metabolic demands (Fortune et al. 2020). Consequently, they exhibit longer life spans (Breed et al. 2025) and longer calving intervals, typically 3–5 years, reflecting the energy demands of their low‐metabolic lifestyle (Best et al. 2001; Burnell 2001; Cooke et al. 2001; Koski et al. 2023). In contrast, rorquals, with their high‐energy lunge‐feeding strategy (Goldbogen et al. 2011), have faster energy recovery rates, supporting shorter calving intervals of 2–3 years (Clapham and Mayo 1990). In line with this, progestogen peaks in the baleen of female humpback whales lasted approximately 10–12 months (Lowe et al. 2022), much shorter than what has been observed in balaenids.

Irrespective of gestation length, calving intervals derived from decades of observational data (e.g., Brandão et al. 2023; Cooke et al. 2001; Davidson et al. 2018; Watson et al. 2021) remain unaffected. However, the length of gestation does influence our understanding of the composition of these intervals—for example, how many months are spent “resting” versus in early pregnancy—and the timing as well as location of conception. From observational data, the common 3‐year calving interval of SRWs is thought to include 1 year each for gestation, lactation, and rest (Best 1994; Best et al. 2001; Cooke et al. 2001; Burnell 2001). This interval length is also reflected in the average reproductive interval inferred from the four baleen plates used in this study (approximately 2.5–3 years). If gestation indeed begins with the rise in progestogens and ends at the second peak, the rest year may be shorter, and gestation longer than previously assumed. This would likely mean conception would occur outside of coastal winter calving grounds. This theory has previously been hinted at by several authors; Payne (1986) and Best (1994) noted that females are rarely seen in mating groups during the year before calving when it is believed they should conceive, and Carroll et al. (2015) and Patenaude et al. (2007) reported increased levels of genetic connectivity between SRW populations that share offshore foraging grounds, possibly indicative of conception at offshore foraging grounds. Such connectivity may increase even further considering the increased sharing of foraging grounds related to climate change alteration of Southern Ocean foraging grounds (ref. Vermeulen, Germishuizen, et al. 2023; Vermeulen et al. 2024).

The glucocorticoid profiles in the baleen plates correlated closely to the progestogen profiles. The correlation between glucocorticoid and progestogen elevations was also observed in NARWs and ascribed to be a normal part of the reproductive cycle (Lysiak et al. 2018). In their review of glucocorticoid patterns across a diverse set of data from different placental mammals, Edwards and Boonstra (2018) explore multiple functions of these hormones during pregnancy, including involvement in initiating parturition, a role in fetal development and organ maturation, and promoting energy mobilization. Future studies should, therefore, aim to better understand the role of glucocorticoids in balaenid species beyond their involvement in the stress response. Although testosterone also seemed correlated with progestogens and estrogens, the general lack of a clear longitudinal pattern limits the formation of clear hypotheses on their role in the reproductive cycle of female SRWs.

In all the above, it remains important to acknowledge that interpretations of the steroid hormone profiles are based on the assumption of a constant baleen growth rate of 27–34 days every 2 cm (Best and Schell 1996), and a constant rate of incorporation of steroid hormones in baleen. However, the physiological demands of migration and gestation could alter baleen growth rates in a way that is not yet known. Sighting histories of photo‐identified NARWs, however, have shown to correspond with certain isotopic signatures in baleen characteristic of the relevant seasonal feeding grounds; these cases, while few, suggest that any seasonal or annual variation in baleen growth rate is likely relatively small (e.g., Hunt et al. 2016). Similarly, annual cycles of bulk stable isotopes in bowhead baleen have generally consistent width across decades, again suggesting that baleen growth rate variation, while it may occur to some extent, is likely not great enough to substantially affect estimates of gestation length.

The temporal resolution of a given point on the baleen could also affect estimates of gestation length. It is not yet clear when and how rapidly circulating progestogens accumulate in growing baleen tissue. As discussed in Hunt et al. (2016), progestogen levels at any point likely represent an average concentration over several weeks or even months rather than a single point in time. Yet, the rapid drop in baleen progestogens documented in pregnant NARWs corresponds tightly with the timing of known births (from sightings dates of neonatal calves), which suggests that hormones derived from a given point on the baleen represent a time period of no more than approximately ~1 month of circulating hormone (Hunt et al. 2016). Similarly, large glucocorticoid peaks corresponding with documented fishing gear entanglements in NARW are often very brief in extent on the baleen, again suggesting a potential temporal resolution of approximately 1 month (Hunt et al. 2016). Although the temporal resolution of the baleen of SRW remains unexamined, we tentatively assume that it may be similar to that seen in NARW.

This paper presents longitudinal profiles of steroid hormone concentrations from the baleen of female SRWs, revealing prolonged progestogen elevations of between 20 and 25 months, likely indicative of a lengthy gestation in the species, and potentially balaenids overall (Hunt et al. 2014, 2016; Lysiak et al. 2018, 2023). While the exact date of fertilization or implantation remains difficult to pinpoint, these findings may have implications for understanding the composition of the 3‐year calving cycle in the species and the timing of mating for conception, relevant in population modeling and regional conservation management strategies. For example, the timing of conception on the summer feeding grounds suggests that some unaccompanied adult SRWs present on the calving grounds in winter may, in fact, be pregnant females. This underscores the importance of minimizing disturbances to these individuals, as early‐stage pregnancy could be particularly sensitive to external stressors. Although assumptions on rates of baleen growth and steroid hormone incorporation introduce some uncertainty, the results highlight knowledge gaps in SRW reproductive physiology and how baleen hormone assessment can act as a powerful tool to address these gaps. This is crucial for developing effective conservation strategies, particularly in light of the climate‐linked demographic changes currently observed in some populations, and for the correct interpretation of how observed elongating calving intervals relate to a physiological response of the species to altered food availability (ref. Vermeulen, Thavar, et al. 2023; Germishuizen et al. 2024). Further research, including multipopulation studies and association with photo‐identification‐based sighting histories, is recommended to refine these estimates.

## Author Contributions

**Loraine Shuttleworth:** data curation (lead), formal analysis (lead), investigation (lead), methodology (lead), writing – original draft (equal). **Andre Ganswindt:** investigation (supporting), methodology (supporting), supervision (equal), validation (lead), writing – review and editing (equal). **Kathleen E. Hunt:** investigation (supporting), methodology (supporting), validation (supporting), writing – review and editing (equal). **Alejandro Fernández Ajó:** investigation (supporting), methodology (supporting), writing – review and editing (equal). **Estefan Pieterse:** formal analysis (supporting), investigation (supporting), methodology (supporting), writing – review and editing (equal). **S. Mduduzi Seakamela:** resources (supporting), writing – review and editing (equal). **Chantel Schoeman:** formal analysis (supporting), investigation (supporting), methodology (supporting), writing – review and editing (equal). **Els Vermeulen:** conceptualization (lead), data curation (supporting), formal analysis (supporting), funding acquisition (lead), investigation (supporting), project administration (lead), resources (lead), supervision (lead), writing – original draft (lead).

## Conflicts of Interest

The authors declare no conflicts of interest.

## Supporting information


Data S1

## Data Availability

The data supporting the findings of this study are available within the article and its Supporting Information. Any other data can be made available upon reasonable request to the corresponding author.
