# Supplementary material for: Multiyear Baleen Endocrine Profiles Suggest a Longer Estimated Gestation in Southern Right Whales (Eubalaena australis)
Source: Ecol Evol. 2025 Jun 17;15(6):e71528. doi: 10.1002/ece3.71528 (PMC12172344; doi:10.1002/ece3.71528)
Supplement: Supplementary file 1 — Data S1 [file ECE3-15-e71528-s001.docx]

**Supplementary material**

**
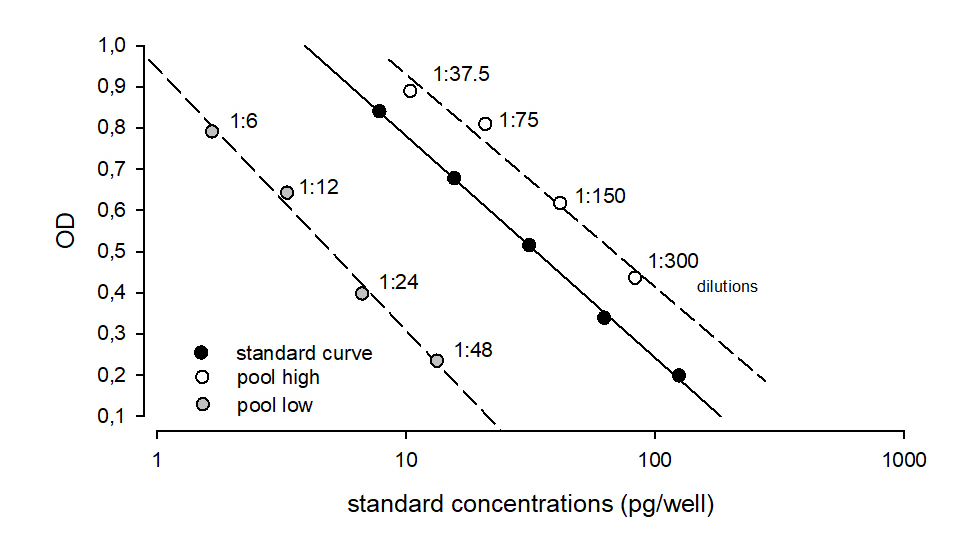
**

**Figure S1** Parallelism test for serial dilutions of high and low sample pools for the selected progestogen enzymeimmunoassay utilizing an antibody against 5β-pregnane-3α-ol-20-one-3HS:BSA.


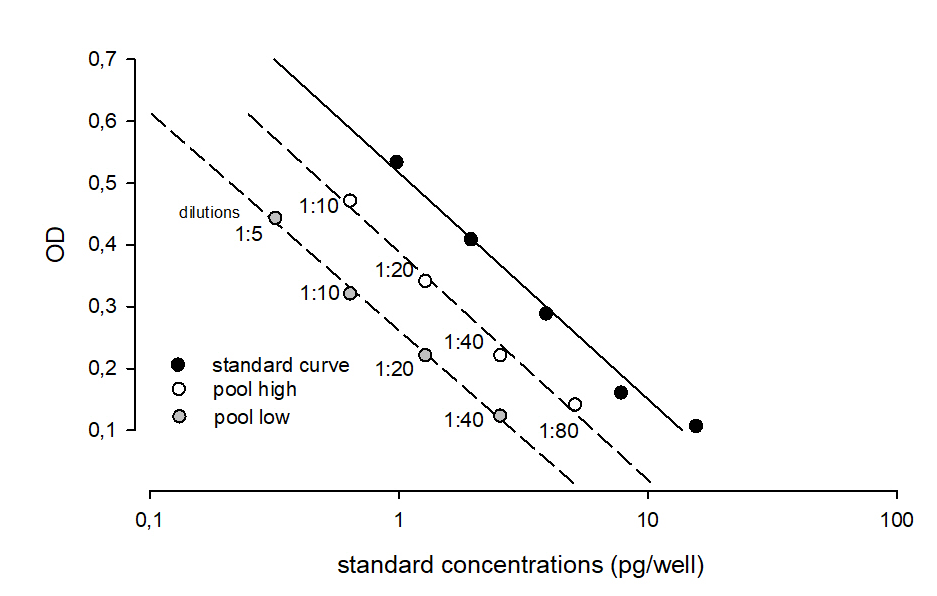


**Figure S2** Parallelism test for serial dilutions of high and low sample pools for the selected estrogen enzymeimmunoassay utilizing an antibody against 17β-oestradiol-17-HS:BSA.


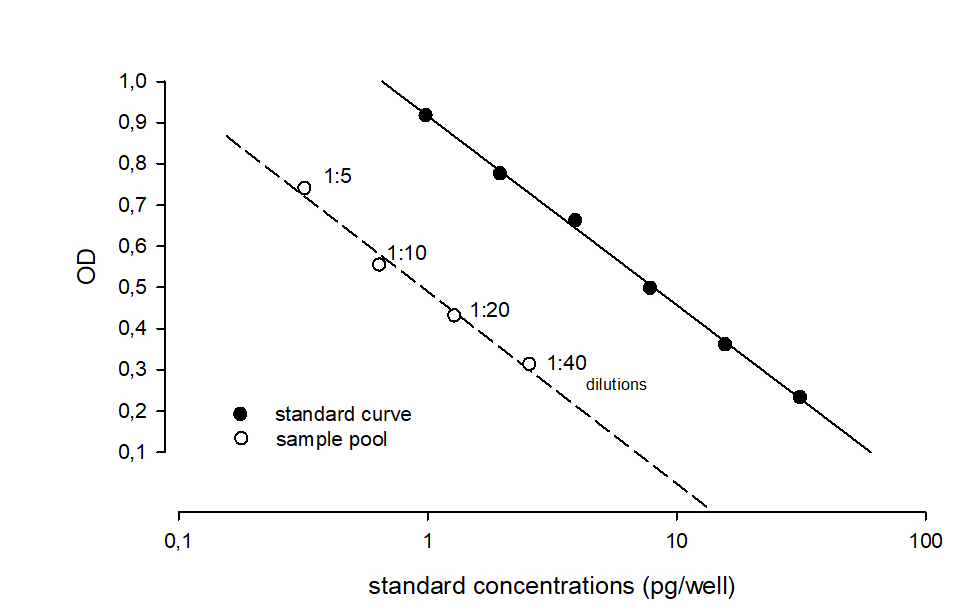


**Figure S3** Parallelism test for serial dilutions of a sample pool for the selected androgen enzymeimmunoassay utilizing an antibody against Testosterone-3-CMO:BSA.

| EIA Name | Label | Antibody raised against | Standard | Sensitivity (ng/g dry weight) | Intra- assay coefficient of variation (%) | | Inter-assay coefficient of variation (%) | |
| --- | --- | --- | --- | --- | --- | --- | --- | --- |
|  |  |  |  |  | **[High] control** | **[Low] control** | **[High] control** | **[Low] control** |
| Cortisol (Palme & Mostl, 1997) | Cortisol-3-CMO- DADOO-biotin | Cortisol-3-CMO-BSA | Cortisol (4-pregnene-11β,17α,21-triol-3,20-dione | 1 | 4.89 | 5.83 | 9.14 | 11.41 |
| Corticosterone (Palme & Mostl, 1997) | Cortisol-3-CMO-DADOO-biotin | Corticosterone-3-CMO-BSA | Corticosterone (4-pregnene-11β,21-diol-3,20-dione) | 3.2 | 2.81 | 6.31 | 7.19 | 9.58 |
| Oxoaetiocholanolone I (Palme & Mostl, 1997) | Cortisol-3-CMO- DADOO-biotin | 11-oxoaetiocholanolone-3-HS:BSA | Cortisol (4-pregnene-11β,17α,21-triol-3,20-dione | 1.6 | 4.95 | 7.46 | 8.51 | 9.20 |
| Oxoaetiocholanolone II (Möstl & Palme, 2002) | 11-oxoaetiocholanolone-17-CMO-biotinyl-3,6,9-trioxaundecanediamin | 11-oxoaetiocholanolone-17-CMO:BSA | 11-oxoaetiocholanolone (5β-androstane-3α-ol-11,17-dione) | 1 | 5.57 | 6.58 | 9.04 | 10.65 |
| 5α-pregnane-3β,11β,21-triol-20-one (Touma et al., 2003) | 5α-pregnane-3β,11β,21-triol-20-one-biotinyl-3,6,9-trioxaundecanediamin | 5α-pregnane-3β,11β,21-triol-20-one –CMO-BSA | 5α-pregnane-3β,11β,21-triol-20-one | 3.2 pe | 4.71 | 5.71 | 6.17 | 6.81 |

**Table S1.** Specifications of the EIAs used to validate suitability for assessing glucocorticoids in baleen powder

*DADOO-biotin = N-biotinyl-1,8-diamino-3,6-dioxaoctane; HS = hemisuccinate; HRP = horseradish peroxidase; CMO = carboxymethyloxime; BSA = bovine serum albumin
